# COVID-19 and Brain Health: Global Council on Brain Health Recommendations

**DOI:** 10.1093/geroni/igab046.078

**Published:** 2021-12-17

**Authors:** Sarah Lock, Lindsay Chura

**Affiliations:** AARP, Washington, District of Columbia, United States

## Abstract

With growing evidence that the coronavirus directly harms the brain and indirectly harms mental well-being due to social isolation and new, increased stressors, the GCBH recognized the urgent need to inform adults age 50+ about ways to their protect brain health as the pandemic continues. In our latest report, the GCBH describes the known neurological symptoms occurring in the short and long term for adults, providing 10 recommendations to protect brain health and urging research in 11 different areas. Calling for an all-of-society approach to protect the brain health of everyone, the GCBH described the negative effects of COVID-19 on people living with Alzheimer’s disease and other dementias and to the impact of health care inequalities. For example, people with dementia were twice as likely to catch the virus as those without dementia; African Americans with dementia had nearly three times the risk of COVID-19 as Caucasians with dementia. The GCBH also points out that caregivers for those living with dementia have experienced particular stress and provided resources and guidance. The Council spotlights the disproportionate toll of COVID-19 on the vulnerable, including racial and ethnic minorities and those living in low- to middle-income countries. After attending this session, participants will be able to identify the neurological impacts of COVID-19, understand the various ways to mitigate risks to brain health, and learn which areas of research will be critical in the future. These recommendations were developed and put forth by the Global Council on Brain Health Governance Committee and Issue Experts.

